# Is ^18^F-FDG-PET/CT an Optimal Imaging Modality for Detecting Immune-Related Adverse Events after Immune-Checkpoint Inhibitor Therapy? Pros and Cons

**DOI:** 10.3390/cancers16111990

**Published:** 2024-05-24

**Authors:** William Karlsen, Lin Akily, Monika Mierzejewska, Jacek Teodorczyk, Artur Bandura, Renata Zaucha, Wojciech Cytawa

**Affiliations:** 1 Students’ Scientific Circle Department of Nuclear Medicine, Medical University of Gdańsk, 80-952 Gdańsk, Poland; wk@gumed.edu.pl (W.K.); linakily@gumed.edu.pl (L.A.); 2 Department of Nuclear Medicine, Medical University of Gdańsk, 80-952 Gdańsk, Poland; m.mierzejewska@gumed.edu.pl (M.M.); teodorczyk@gumed.edu.pl (J.T.); 3 Department of Clinical Oncology and Radiotherapy, Medical University of Gdańsk, 80-952 Gdańsk, Poland; abandura@gumed.edu.pl (A.B.); rzaucha@gumed.edu.pl (R.Z.)

**Keywords:** immunotherapy, immune checkpoint inhibitors, immune-related adverse events, ^18^F-FDG PET/CT, metabolic activation patterns

## Abstract

**Simple Summary:**

Recent advances in cancer therapy have spotlighted immune checkpoint inhibitors (ICIs) as a breakthrough in treating various cancers. These treatments, however, can lead to immune-related adverse events (irAEs) that mirror the body’s heightened immune response, affecting multiple organs. Recognizing and managing these irAEs is critical, and imaging modalities such as ^18^F-FDG PET/CT have emerged as a potential tool for early detection. This review delves into the capability of ^18^F-FDG PET/CT to identify irAEs, exploring the patterns of metabolic activation indicative of these events. While highlighting the current utility of PET/CT scans in oncology for tracking therapy response and irAEs, the review also speculates on future directions, suggesting a potential role in refining immunotherapy strategies and enhancing patient care. The insights obtained could transform patient monitoring during ICI therapy, potentially improving outcomes by facilitating prompt management of irAEs.

**Abstract:**

Immunotherapy with immune checkpoint inhibitors (ICIs) has revolutionized contemporary oncology, presenting efficacy in various solid tumors and lymphomas. However, ICIs may potentially overstimulate the immune system, leading to immune-related adverse events (irAEs). IrAEs may affect multiple organs, such as the colon, stomach, small intestine, kidneys, skin, lungs, joints, liver, lymph nodes, bone marrow, brain, heart, and endocrine glands (e.g., pancreas, thyroid, or adrenal glands), exhibiting autoimmune inflammation. ^18^F-fluorodeoxyglucose positron emission tomography/computed tomography (^18^F-FDG PET/CT) is commonly used in oncology for staging and assessment of therapy responses, but it may also serve as a tool for detecting irAEs. This review aims to present various patterns of metabolic activation associated with irAEs due to ICI treatment, identifiable through ^18^F-FDG PET/CT. It describes the advantages of early detection of irAEs, but also presents the challenges in differentiating them from tumor progression. It also delves into aspects of molecular response assessment within the context of pseudoprogression and hyperprogression, along with typical imaging findings related to these phenomena. Lastly, it summarizes the role of functional PET imaging in oncological immunotherapy, speculating on its future significance and limitations.

## 1. Introduction

Immunotherapy using immune checkpoint inhibitors (ICIs) has transformed contemporary oncology, demonstrating efficacy across various solid tumors and lymphomas. Within the tumor microenvironment, specific monoclonal antibodies representing ICIs play a crucial role in reversing T-cell exhaustion and enhancing the body’s natural defense against tumor cells [1,2].

Numerous pathways, including CTLA-4 (cytotoxic T-lymphocyte-associated protein 4), PD-1/PDL-1 (programmed cell death protein-1), TIGIT (T-cell immunoreceptor with immunoglobulin and ITIM domain), HLA-G (human leukocyte antigen G), TIM-3 (T-cell immunoglobulin domain and mucin domain 3), KIR (killer Ig-like receptors), and CD137, have been investigated in clinical trials. Currently, two main pathways are widely employed in clinical practice—one targeting CTLA-4 and the other targeting PD-1 or PD-L1. Inhibitors of CTLA-4 and PD-1/PD-L1 have shown success in various cancers, such as melanoma, non-small cell lung cancer (NSCLC), head and neck squamous cell carcinoma (HNSSC), urothelial carcinoma, colorectal cancer, cervical cancer, endometrial carcinoma, triple-negative breast cancer (TNBC), renal cell carcinoma (RCC), hepatocellular carcinoma (HCC), or Hodgkin lymphoma (HL), producing promising results [3,4].

However, ICIs may potentially overstimulate the immune system, leading to immune-related adverse events (irAEs). Possible mechanisms of irAEs are co-existence of T-cell activity against tumor and healthy tissues (shared antigens), increased levels of pre-existing host autoantibodies and inflammatory cytokines, or intensification of complement-mediated inflammation [3]. Their symptoms vary depending on multiple factors, such as the type of ICIs, tumor type and location, and patient innate susceptibility. IrAEs may affect various organs, such as the colon, stomach, small intestine, kidneys, skin, lungs, joints, liver, lymph nodes, bone marrow, brain, heart, and endocrine glands (e.g., pancreas, thyroid, or adrenal glands), exhibiting autoimmune inflammation [4,5,6,7]. Involvement of hormone-producing organs may lead to conditions such as hypo/hyperthyroidism, pituitary insufficiency, hypocortisolism, or diabetes.

IrAEs range from mild to life-threatening in ICI therapy. Management of irAEs is contingent on their severity, with treatment being temporally or permanently discontinued based on the guidelines of oncological organizations such as ESMO [8]. For certain irAEs such as colitis or pneumonitis, treatment must be stopped, but in most instances, it can be continued, possibly indicating a favorable prognosis, particularly, if the patient experiences irAE of low grade [9,10,11]. Recent observational studies and meta-analyses demonstrate a positive correlation between irAEs and improved progression-free survival (PFS) [3,12,13].

^18^F-fluorodeoxyglucose positron emission tomography/computed tomography (^18^F-FDG PET/CT) is commonly used in oncology based on the increased glucose metabolism present in most neoplasms. However, the non-specific character of ^18^F-FDG also allows visualization of inflammatory changes, including autoimmune reactions. Consequently, ^18^F-FDG PET/CT may serve as a tool for detecting irAEs.

This article aims to present various patterns of metabolic activation associated with irAEs identifiable through ^18^F-FDG PET/CT. It also explores molecular response assessment in the context of pseudoprogression and hyperprogression, along with a comment on the typical imaging findings related to these phenomena. As compared to the existing literature, our review broadly covers the issues of PET identification and monitoring of various irAEs that may occur across multiple organs. We detail organ-specific metabolic changes, provide quantitative parameters of PET diagnostics (e.g., SUVmax or time to peak irAE intensity), and discuss the implications of these findings for patient management. We support the discussion with relevant clinical cases, showing the complexity and difficulty in imaging responses to immunotherapy. Lastly, we summarize the role of functional PET imaging in oncological immunotherapy, speculating on its future significance, development, and limitations.

## 2. Materials and Methods

To build the body of literature for our review, we systematically searched on PubMed for articles from 2019 to December 2023, using the following keywords and appropriate Boolean operators: (“1 January 2019”[Date–Create]: “31 December 2023”[Date–Create]) AND ((“Disease Progression”[Mesh] OR “hyperprogression”[All Fields] OR “pseudoprogression”[All Fields]) OR (“Immune Checkpoint Inhibitor Related Adverse Effects”[All Fields]) OR (“Programmed Cell Death 1 Receptor”[Mesh] OR “programmed cell death 1 receptor”[All Fields]) OR (“CTLA-4 Antigen”[Mesh] OR “CTLA-4 Antigen”[All Fields]) OR (“PDCD1 protein, human”[All Fields]) OR (“nivolumab”[All Fields] OR “pembrolizumab”[All Fields] OR “durvalumab”[All Fields] OR “ipilimumab”[All Fields] OR “avelumab”[All Fields] OR “atezolizumab”[All Fields])) OR ((“immune-related adverse events”[All Fields] OR “IRAE”[All Fields] OR “immune checkpoint inhibitors”[All Fields])) AND ((“FDG-PET”[All Fields] OR “FDG positron emission tomography”[All Fields] OR “18F-FDG positron emission tomography”[All Fields])). In this review, we have chosen to focus primarily on literature published after 2019 to ensure the most up-to-date understanding of ICI-related irAEs. This decision is based on rapid progress in the field of immunotherapy, where recent studies frequently update and refine our knowledge regarding the efficacy, safety, and mechanistic insights of ICIs. Moreover, according to PubMed statistics after 2019, there was a rapid increase in publications concerning irAEs following ICI treatment.

**Figure 1:**
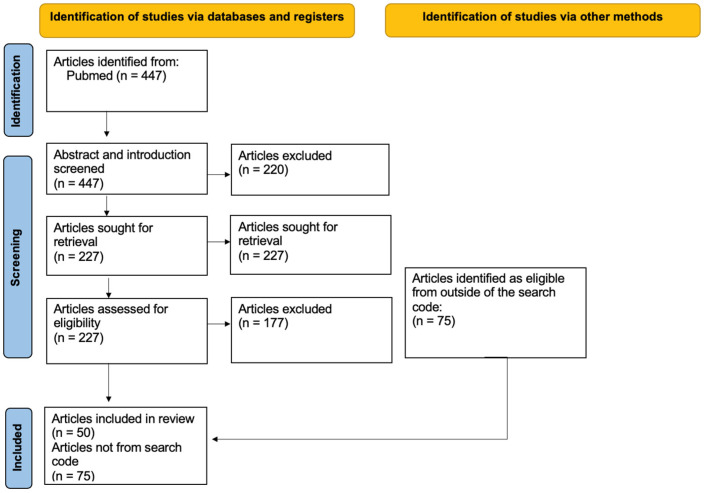
A flow diagram of the search procedure for the narrative review.

Initially, 447 articles were identified through this search, of which 50 were ultimately considered eligible and included in the study, as shown in Figure 1. The inclusion criteria were:

- IrAEs diagnosed by ^18^F-FDG PET/CT
- IrAEs caused by ICIs
- ICI as treatment

Exclusion criteria were:

- If the pathology is not caused by ICI (irAEs caused by other forms of immunotherapy were rejected)
- Article published before 2019

While writing the manuscript, we found an additional 75 articles, mainly through the references to the initial articles coming from the search code. We decided to cite them because they enriched the review (outside the search code, Figure 1).

## 3. Immune Checkpoint Inhibitors

ICIs, a group of anticancer drugs introduced into oncological clinical practice roughly a decade ago, have demonstrated remarkable effectiveness in numerous clinical trials, with sustained responses and prolonged survival. By inhibiting specific interactions between immune cells and cancer cells, they remove obstacles to the immune system’s defense against cancer. For the discovery of the two pathways of ICIs, i.e., CTLA-4 and PD-1/PD-L1, two researchers, Tasuku Honjo and James Allison, respectively, were awarded the Nobel Prize in physiology or medicine in 2018 [14].

**Figure 2:**
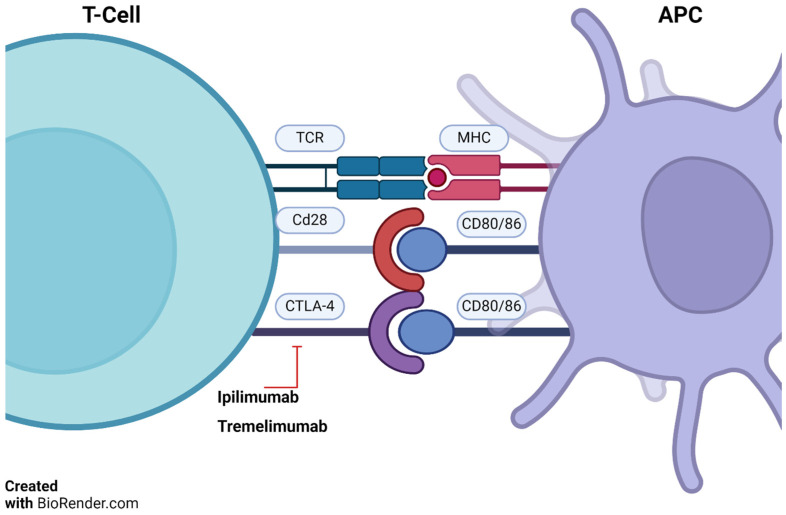
T-cell/APC interaction and ipilimumab action. TCRs (T-cell receptors) bind antigen presented by antigen presenting cells (APC)—via major histocompatibility complex (MHC). CD28 on T-cells and CD80/86 on APCs provide necessary co-stimulation. Ipilimumab blocks cytotoxic T-lymphocyte-associated protein 4 (CTLA-4), boosting anti-tumor immunity. Adapted from Basudan, 2022 [14,15]. BioRender.com accessed on 4 December 2023.

CTLA-4 is a protein on T-cells that helps control the immune system’s response to antigens. It competes with CD28, a protein that activates T-cells. Ipilimumab and tremelimumab inhibit CTLA-4, allowing CD28 to activate T-cells (Figure 2) [14,15].

**Figure 3:**
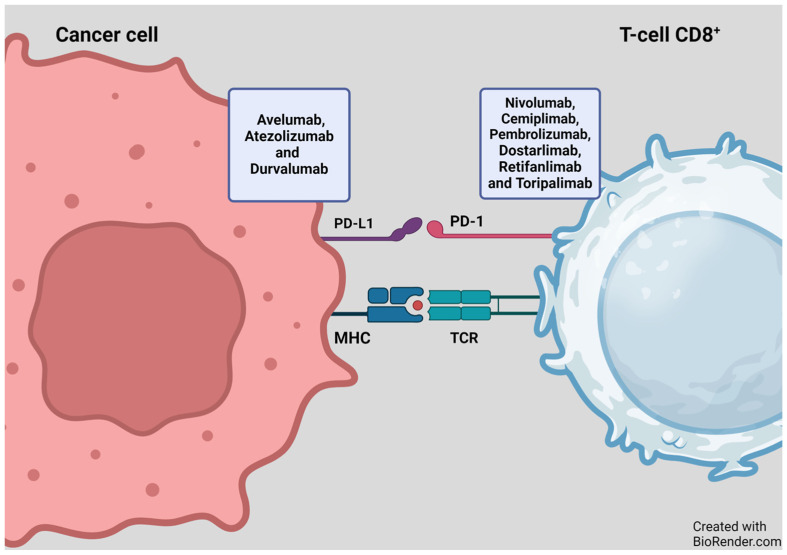
Mechanism of action of PD-1/PD-L1 inhibitors. Figure adapted from Basudan, 2022 [14,15]. MHC—major histocompatibility complex, programmed cell death protein-1 (PD-1) or its ligand-1 (PD-L1). BioRender.com accessed on 4 December 2023.

**Figure 4:**
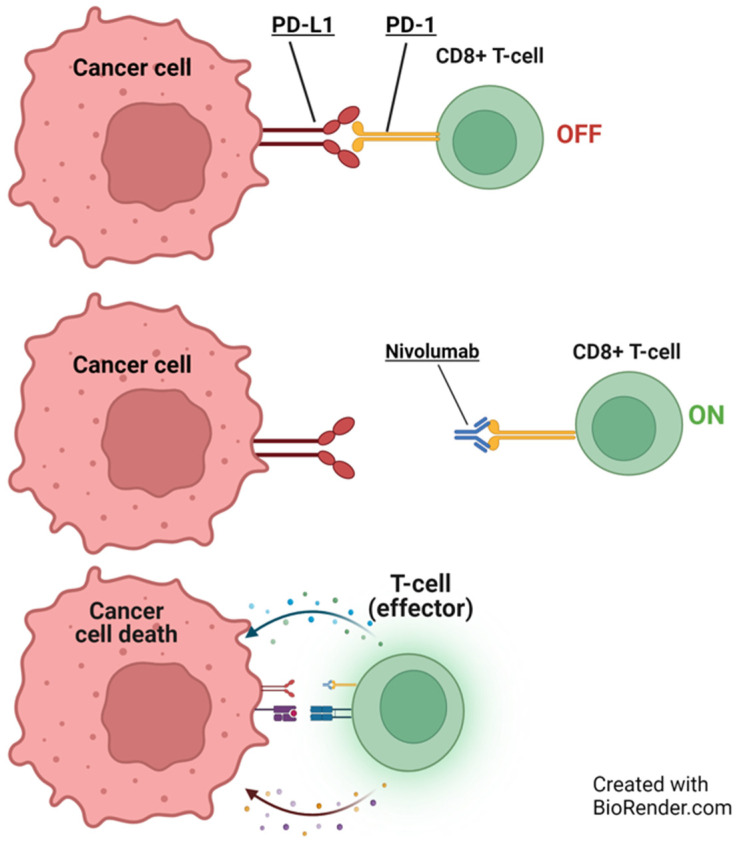
Illustration of nivolumab’s role in cancer immunotherapy. Initially, PD-L1 on a cancer cell deactivates a CD8+ T cell (O‘FF’). Nivolumab blocks programmed cell death protein-1 (PD-1), activating the T-cell (O‘N’), leading to cancer cell death. Adapted from Mayoral et al., 2019 [16]. BioRender.com accessed on 4 December 2023.

PD-1, an inhibitory receptor present on all activated T-cells, serves as a regulator of T-cell effector function and aids in maintaining the balance of immune reactions. On the other hand, PD-L1 is present on some cancer cells and normal immune cells. By binding to PD-1, PDL-1 dampens the activity of T-cells, which play a role in regulating immune responses. ICIs specifically target this interaction, preventing PD-1 from binding to PD-L1. This interference allows T-cells to exert a more potent attack on cancer cells (Figure 3). ICIs such as pembrolizumab, nivolumab, cemiplimab, dostarlimab, retifanlimab, and toripalimab attach to the PD-1 protein on T-cells. In contrast, atezolizumab, durvalumab, and avelumab bind to the PD-L1 receptor on cancer cells (Figure 3 and Figure 4) [14,15].

## 4. Malignancies Commonly Treated with ICIs

ICIs are nowadays commonly used in multiple malignancies. In advanced melanoma, ipilimumab combined with nivolumab gives 50% five-year overall survival (OS) [17,18]. In NSCLC, five-year OS increased from 14% to 23.7%. ICIs are also effective in advanced small cell lung cancer (SCLC) [19]. Even in tumors with very low tumor mutation burden (TMB = 1 mutation per megabase), such as clear cell renal cancer, nivolumab plus ipilimumab fared significantly better compared to other treatment methods [20]. Nivolumab and pembrolizumab are also approved for the treatment of HNSCC [21], locally advanced or metastatic urothelial carcinoma, colorectal cancer, gastric cancer, and many others with proven microsatellite instability [3,22,23]. Atezolizumab plus bevacizumab (angiogenesis inhibitor) is a choice for unresectable HCC [22]. Atezolizumab plus paclitaxel is recommended for PD-L1-positive TNBC [15,24,25]. Nivolumab and pembrolizumab are effective in HL [26]. Avelumab is indicated for the treatment of Merkel cell cancer. An overview of common indications for ICIs is presented in Table 1.

## 5. Molecular Response Assessment in FDG-PET in the Context of ICIs Treatment

The addition of functional information, such as glucose metabolism, can significantly improve the assessment of responses. The most spectacular example of functional imaging incorporated into clinical management protocol was HL, where the FDG PET interpreted using the Deauville score brought a “paradigm shift” in response evaluation [40]. Metabolic assessment is also used in non-Hodgkin’s lymphomas (NHL), provided they present with significantly high glucose uptake (majority of NHL). In several solid tumors, such as breast cancer, NSCLC, esophageal cancer, and Ewing sarcoma, FDG PET has also been superior at response evaluation compared to pure anatomical imaging [41,42,43,44,45]. The classical PET-based criteria to assess metabolic response in solid tumors—EORTC (European Organization for Research and Treatment of Cancer) proposed in 1999 and PERCIST (PET Response Criteria in Solid Tumors) proposed by Wahl et al. in 2009 [46,47]—did not refer to immunotherapy. Recently, a few more criteria were established to adjust the treatment response to immunotherapy. These are: PECRIT (PET/CT Criteria for Early Prediction of Response to Checkpoint Inhibitor Therapy) [48], PERCIMT (PET Response Evaluation Criteria for Immunotherapy) [49], imPERCIST (Immunotherapy-modified PERCIST) [50], and iPERCIST (immune PET Response Criteria in Solid Tumors) [51].

While using FDG PET/CT in response assessment for ICIs, a few critical aspects should be discussed. First, by its nature, a consequence of ICIs treatment is an intensification of T lymphocyte infiltration (so-called tumor-infiltrating lymphocytes, TILs) into the tumor microenvironment, which may result in its paradoxic growth and metabolic activation. In this context, a pseudoprogression should not surprise; however, the criteria should consider the right moment for a reliable response assessment [52]. Second, due to overstimulation of the immune system by ICIs, various irAEs may appear (as mentioned above), and a reading nuclear medicine physician should be aware of their possible metabolic patterns in PET imaging, to minimize the risk of false-positive results. Last, the interpretation of metabolic changes in the follow-up PET scan should not only focus on the tumor and the organs typically related to irAEs but also analyze other locations, e.g., spleen, where the change in uptake may indirectly predict the response to treatment [53].

Metabolic patterns of typical irAEs and challenges of metabolic imaging in patients receiving ICIs are presented in the following paragraph and summarized in Table 2.

## 6. IrAEs Caused by Immune Checkpoint Inhibitors and Their Manifestation in FDG-PET

### 6.1. General Endocrine

**Figure 5:**
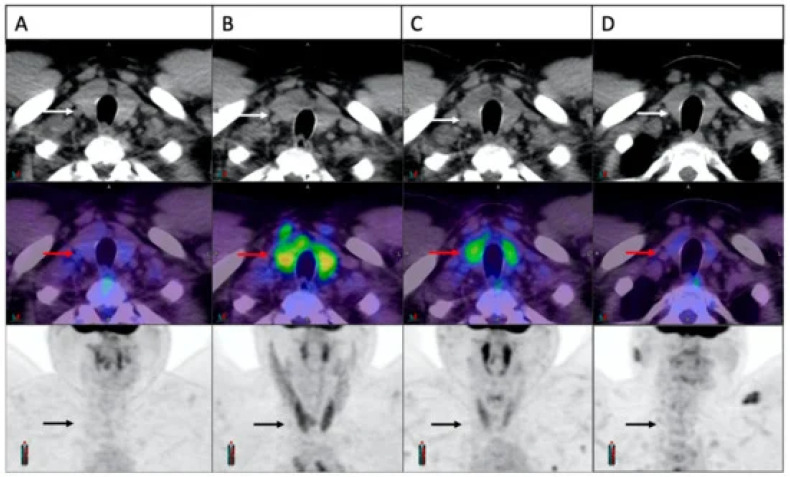
Four FDG-PET scans showing a patient with ICI-induced thyroiditis and the evolution of thyroid FDG uptake over time. Initially, the thyroid appeared normal ((**A**), arrows). Three weeks into treatment, there was a notable rise in FDG uptake ((**B**), arrows), followed by partial resolution at 6 weeks ((**C**), arrows) and complete resolution at 24 weeks ((**D**), arrows). The material was originally published in Galligan et al. (2023) [56].

ICIs can lead to endocrine irAEs, with PET/CT scans showing abnormal FDG uptake in the thyroid, pituitary, and pancreas indicating thyroiditis, hypophysitis, and pancreatitis, respectively [54]. Hypophysitis incidence ranges from 3.25% to 14.0%, mainly from anti-CTLA4 treatments, and it is detectable by increased pituitary-to-frontal SUVmax on PET scans [55,56]. Adrenal insufficiency occurs in 2.43% of ICI-treated patients, especially with anti-CTLA-4 [57]. Thyroid dysfunction, most often hypothyroidism, after ICI therapy, correlates with a diffuse increase in thyroid FDG uptake, peaking around day 42 [6,56,58,59,60]. While current evidence predominantly indicates diffuse FDG uptake in the thyroid and adrenal glands during irAEs, focal uptake analogous to observations in the pancreas and lungs cannot be completely ruled out. Focal uptake is commonly more consistent with thyroid cancer [61]. However, such focal presentations related to irAE may be underreported due to the subtlety of their appearance or the stage at which they appear. Although PET/CT is not indicated to monitor for thyroid dysfunction during immunotherapy, accidental findings that may be present in the scans should always be noted and investigated for patient benefit [62] (Figure 5). In cases of suspected ICI-related thyroiditis where the use of PET is not possible or is not giving a clear result, we can use radioiodine scan, although its pattern varies from PET. In cases of destructive thyroiditis, there is a low uptake of radioiodine, resulting in low/lacking visibility on the scan [63]. An alternative option available is a technetium-99m thyroid scan, which basically presents similar patterns to a radioiodine scan [64].

PET/CT can detect and monitor endocrine irAEs such as thyroiditis, which occurs in melanoma patients on pembrolizumab, preparing clinicians for potential thyroid hormone replacement [56,65,66]. ICIs may also cause hypercalcemia and rare events such as hypoleptinemia, which cause lipodystrophy [67,68]. Current evidence does not provide definitive guidance on the optimal timing for conducting scans to detect irAEs in these organs. Due to the variability in the onset and progression of irAEs, as well as individual patient responses to ICI therapy, it remains challenging to pinpoint the exact timing for imaging assessments; however, according to Nobashi et al. [60], the maximum uptake in the thyroid is expected to be on day 142. Further research would be warranted to establish more precise timelines that could enhance the accuracy and effectiveness of monitoring these events.

On some rare occasions, adrenalitis occurs as an irAE; on PET, it is mainly seen as increased FDG uptake; when turning into a chronic phase, it may be seen as bilateral atrophy of the glands [69].

### 6.2. Hepatitis

Severe immune-related hepatitis occurs in 1–11% of patients. It commonly presents as a rapid increase in liver enzymes and/or bilirubin level. If not treated, this condition may cause the patient’s death. Cases with pre-existing liver steatosis or cirrhosis may pose a diagnostic dilemma. An increased liver-to-blood-pool SUVmean ratio (1.3–1.4 in healthy individuals) helps in identifying hepatitis, which usually presents as diffusely increased parenchymal metabolic activity [6,69,70]. However, early hepatitis may not be visible on PET images due to physiological liver uptake. Unlike hepatitis, liver metastases typically present with focally increased uptake [54].

### 6.3. Splenitis

A few studies linked the high splenic-to-liver ratio on PET/CT to splenic irAEs, notably with ipilimumab in metastatic melanoma [7,71,72]. However, splenic irAEs are relatively rare, and splenic uptake is more often regarded as a surrogate of immune system activation and thus a potential predictor of response to immunotherapy [54]. In a cohort of 43 patients with stage III NSCLC treated with chemoradiotherapy, of whom 16 had additional durvalumab maintenance treatment, an increase in FDG uptake in the spleen by 12.5% was observed in the ICI group (with significantly longer OS), as opposed to the controls, who had a reduction of splenic activity (−4.4%) [73]. On the other hand, Prigent et al. (2021) noted that a >25% increase in the spleen-to-liver ratio in melanoma patients at 3 months of treatment with ICI indicates a poorer prognosis [53]. Also, some patients may develop hemolytic anemia as an irAE, secondarily causing increased splenic uptake of FDG, which should be considered a differential diagnosis of ICI-splenitis [10]. Therefore, splenic uptake during immunotherapy with ICIs should be interpreted with caution and in close correlation with clinical parameters.

### 6.4. Pancreatitis

ICI-induced pancreatitis is a rare irAE, accounting for <1% of events [74]. ICI may cause injury to both endocrine and exocrine pancreatic function. Usually, diabetes occurs later than pancreatitis, suggesting that endocrine dysfunction might be a complication of pre-existing pancreatitis [75].

The reported radiological characteristics of acute pancreatitis caused by ICIs may include enlargement of the pancreas, accumulation of fluid, uneven enhancement, necrosis, formation of cysts, calcification, and shrinkage [11,76]. Twenty-five patients with ICI-pancreatitis with elevated serum lipase/amylase caused by anti-PD1/PD-L1 drugs were studied by Das et al. (2020). Two out of three (66%) patients who had PET scans presented abnormally increased FDG uptake [76,77].

Acute ICI-pancreatitis may also present as focal changes in FDG uptake mimicking metastases [11]. According to Berz et al. (2023), pancreatitis is primarily observed in combination therapy with nivolumab and ipilimumab [7]. Also, atrophic exocrine pancreatic insufficiency was seen with anti-PD1 therapy [78]. To sum up, any case of abnormal FDG uptake in the pancreas, both diffuse and focal, should be evaluated thoroughly.

### 6.5. Pneumonitis

Pneumonitis is a rare but sometimes lethal irAE following ICIs. It is even the most lethal irAE with anti-PD-1/PD-L1 drugs, being the cause of ~35% of immunotherapy-related deaths (~1% of deaths by CTLA-4 inhibitors), making it sometimes necessary to discontinue treatment [7,29,79,80]. Nishino et al. (2016) found that the median onset time of pneumonitis was 2.6 months after immunotherapy initiation [81]. 

**Figure 6:**
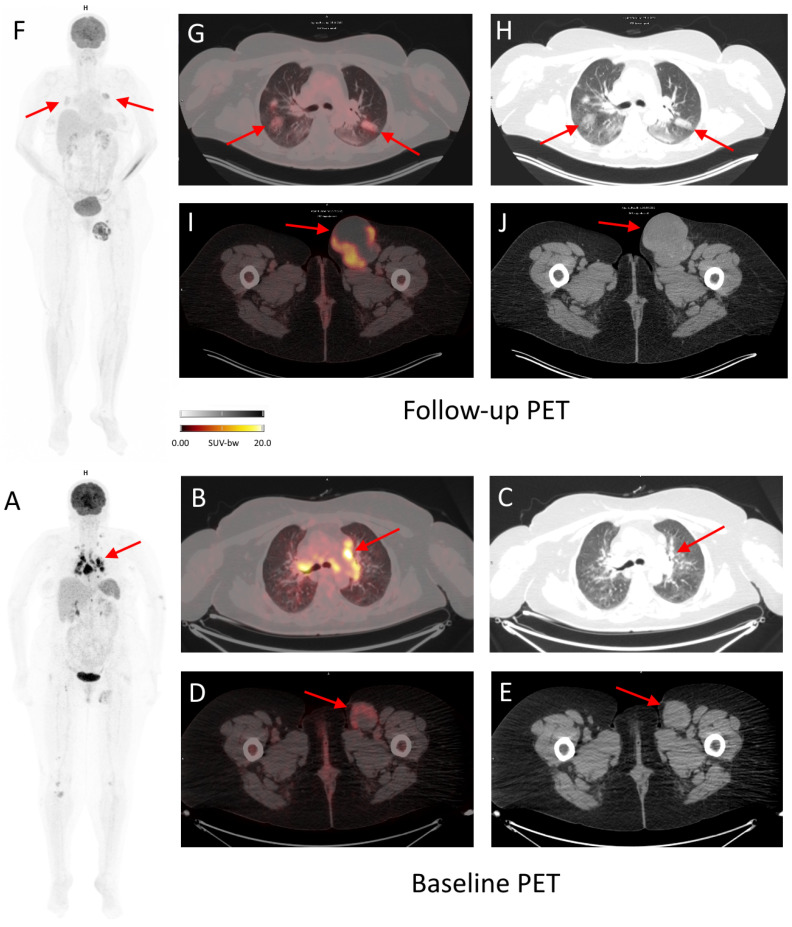
Baseline and follow-up ^18^F-FDG PET/CT of a 52-year-old woman with inoperable, stage IV melanoma treated with cemiplimab for 7 months. During the course of the treatment, the patient was diagnosed with immunotherapy-related pneumonia, grade 2, seen as metabolically active (SUVmax 7.3) ground glass opacity changes in both lungs ((**F**–**H**), arrows). The patient began treatment with prednisone (50 mg/day) with good radiographic response. The follow-up PET scan also revealed complete metabolic response of majority of pathological lymph nodes (seen in baseline PET, e.g., mediastinal, (**A**–**C**) arrows), except for the left inguinal lymph nodes, where progression was observed ((**I**,**J**) in follow-up PET vs. (**D**,**E**) in baseline PET; arrows), which could suggest dissociated response. However, due to previous immunotherapy with nivolumab and ipilimumab (before baseline PET), pathological lymph nodes in mediastinum should be differentiated between metastatic and due to sarcoid-like reaction.

PET findings in ICI-pneumonitis include bilateral heightened FDG uptake in the lungs, typically presenting as interstitial or peribronchovascular consolidations that may manifest as organizing pneumonia in focal patterns or be associated with diffuse ground glass opacities [11,29,54,82,83]. It must be stressed that PET findings are non-specific, making it necessary to consider the clinical situation to make a proper diagnosis [7]. Some patients present only bronchial wall thickening, micronodules, and/or symptoms such as asthma. In the lung parenchyma of patients with ICI-pneumonitis, the mean metabolic activity reaches an SUVmax of 3.36 (+/− 1.7) [80]. An example of ICI-pneumonitis is presented in Figure 6.

Special consideration is needed for lung cancer, the leading cause of cancer mortality worldwide. Nowadays, adjuvant ICI after radical radiochemotherapy (RTCHT) for the chest is a standard of care in the treatment of stage III NSCLC [84]. ICI (with or without chemotherapy) is also used for the treatment of stage IV NSCLC and extensive disease (in combination with chemotherapy) in SCLC, both sometimes requiring palliative radiotherapy to the chest [85,86]. As radiotherapy alone as well as ICI might lead to pneumonitis, distinguishing between those two causes of lung toxicity is challenging. Moreover, lung cancer patients often have some pre-existing lung disease. In PACIFIC, a registration trial for consolidative durvalumab after RTCHT in stage III NSCLC patients, any-grade pneumonitis occurred in 33.6% on durvalumab and 24.9% on the placebo arm [87]. Usually, immune-related pneumonitis is predominant in the lung interstitium, does not cross lung fissures, and occurs after several therapies, whereas radiation pneumonitis is observed at the edge of the radiation field (may cross lung fissures) and occurs less than 6 months after completion of RTCHT [88]. ICI may also lead to recall pneumonitis in irradiated patients (in about 7%) [89]. Hence, ICI-related pneumonitis is a diagnosis of exclusion [88].

Pneumonitis is graded by symptoms and radiographical signs. To the best of the authors’ knowledge, today FDG PET is not used for grading irAEs; however, it could play an important role in assessing and grading irAEs in the future [55].

### 6.6. Sarcoid-like Reaction

**Figure 7:**
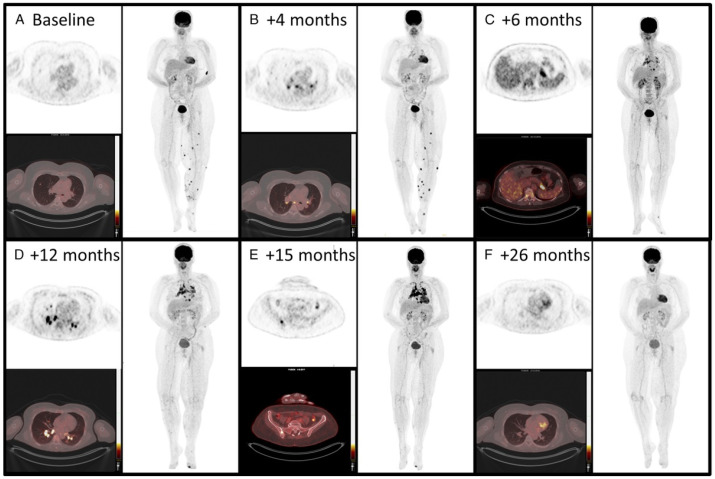
A 50-year-old woman with metastatic melanoma treated with pembrolizumab showed evolving ^18^F-FDG PET/CT findings. Initially (**A**), scans showed lung and left leg lesions, considered metastatic. At 4 months (**B**), FDG uptake appeared in mediastinal and hilar lymph nodes, and by 6 months (**C**), it extended to the spleen and pancreatic tail. By 12 months (**D**), the sarcoid-like reaction progressed to additional lymph nodes. At 15 months (**E**), involvement extended to the thoracic vertebra and left iliac bone, confirmed as granulomatous inflammation. After stopping pembrolizumab (**F**), all FDG-avid lesions resolved, illustrating the need to differentiate sarcoid-like reactions from disease progression in immunotherapy. The material was originally published in van Willigen et al. (2019) [92]; with permission from Wolters Kluwer.

Up to 7% of patients using ICIs develop a sarcoid-like reaction, which is the development of non-caseating epithelioid cell granulomas without fulfilling the criteria of systemic sarcoidosis. Typically, it presents as FDG-avid lymph nodes or lesions in the lungs, skin, and rarely bone or upper abdomen, mainly hepatic hilum or spleen [6,10,11,90]. FDG PET usually shows bilateral, symmetric, hypermetabolic mediastinal and hilar lymphadenopathy with or without hypermetabolic focal nodular opacities or consolidations in the lungs. Enhanced FDG uptake in subcutaneous hypermetabolic nodules due to non-caseating granulomas might also be seen in sarcoid-like reactions. It may present with typical symptoms of sarcoidosis, such as wheezing, chest pain, dyspnea, or a non-productive cough, and clinical signs, such as increased angiotensin-converting enzyme, hypercalcemia, and increased liver enzymes [6,83,91]. Although FDG PET is a sensitive method for detecting sarcoid-like lesions, it still lacks specificity, and these lesions may mimic metastatic disease. Hence, in equivocal cases, a biopsy should be performed to exclude cancer progression. After completion of ICI treatment or in response to steroids, sarcoid-like lesions should resolve (Figure 7).

### 6.7. Gastritis

FDG PET/CT imaging often reveals increased metabolic activity, diffused or localized, in the stomach lining, indicating inflammation. Gastritis can be asymptomatic or symptomatic, necessitating further investigation in endoscopy [74]. Interestingly, gastritis detected during interim PET/CT in about 20% of patients treated with immunotherapy for NSCLC might be a novel imaging biomarker of better survival [93].

### 6.8. Esophagitis

Esophagitis can be identified on PET/CT scans as diffuse FDG uptake along the esophageal tract, which may correlate with symptoms such as dysphagia or odynophagia [54]. Duodenitis and ileitis are rare irAEs that also have been seen on FDG PET [54].

### 6.9. Colitis

Colitis, mainly following anti-CTLA-4 ICI, is an irAE with the highest fatality following anti-CTLA-4 drugs, according to Léger et al. [11,29]. The potentially severe course makes it necessary to consider stopping treatment and/or prescribing corticosteroids [79]. In 10% of patients with severe ICI-colitis under combination treatment of anti-CTLA-4 and anti-PD-1, hospitalization was necessary, as reported by Huff et al. [94]. A mild presentation of immune-related colitis in the form of diarrhea is common, occurring in up to 49% of patients receiving high doses of ipilimumab [58,89].

**Figure 8:**
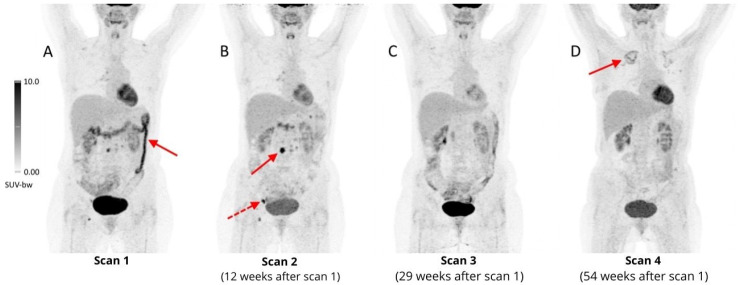
Four follow-up ^18^F-FDG PET/CT scans of a 41-year-old woman with refractory HL during the fourth line of treatment, with nivolumab. The first PET scan performed less than one year after initiation of immunotherapy revealed increased, diffuse metabolic activity (SUV_max_ 11.6) in the transverse and descending colon, suggesting ICI-colitis ((**A**), arrow), which resolved after completion of nivolumab treatment 12 weeks after the first scan (**B**). During the treatment some involved lymph nodes presented with transiently increased ^18^F-FDG uptake, e.g., periaortic ((**B**), arrow, SUV_max_ 15.3) and right inguinal ((**B**), dashed arrow, SUV_max_ 13.5), with a complete metabolic response in the following PET scan (**C**), which should be considered as pseudoprogression. A month after the 3rd scan, an allogeneic stem cell transplant was performed, complicated by acute graft-versus-host disease, which manifested as grade III colitis. After administration of vedolizumab (IgG1 antibody, α4β7 integrin antagonist, highly selective inhibitor of gastrointestinal tract inflammation), a complete remission of inflammatory changes in the colon was achieved (**D**). In the last follow-up scan, diffuse areas of mildly increased FDG uptake were reported in the upper lobes of both lungs (more prominent on the right side), which were diagnosed as aspergillosis ((**D**), arrow).

Lang et al. [95] stated that diffuse thickened colon, a radiological hallmark of colitis, was only seen in 5% of patients studied by CT (6 out of 119), leaving a large room for error, while PET findings significantly correlated with clinically significant diarrhea. This means that ICI-colitis should not be diagnosed with CT, and that FDG PET is more sensitive for detection of colitis (example in Figure 8). It is also important to refer the results to the clinical picture and not rely solely on PET for diagnosis, as this could potentially lead to premature discontinuation of treatment. Another article by Schierz et al. [6] states that on unenhanced CT, as a part of PET/CT, colonic wall thickening might be seen with pericolic fat stranding and segmental or diffuse increased FDG uptake [6]. Cho et al. mention that increased uptake of FDG in the colon with concurring diarrhea was present in 49% of their 100 patients treated with ipilimumab [83]. The peak day for SUVmax is on day 283, just like in pancreatitis [60].

FDG PET interpretation should include information about the usage of anti-diabetic drugs (mainly metformin), since they are known to cause non-specific activation of the intestinal tract. In some cases, it may be justified to withdraw metformin 48 h before imaging [17].

### 6.10. Nephritis

IrAEs concerning kidneys most commonly present as acute kidney injury in the form of acute tubulointerstitial nephritis [7,54]. They are mainly diagnosed by laboratory tests but could be a spontaneous finding on follow-ups with PET [79]. FDG PET/CT generally presents a diffuse bilateral uptake in the renal cortex, with >30% kidney enlargement [11,96]. A renal parenchymal SUVmax-to-blood pool ratio is increased compared to baseline, while the renal pelvis SUVmax-to-blood pool ratio is typically significantly lower at nephritis compared to baseline [96]. Importantly, nephritis is one of the few irAEs that might be delayed more than 3 months, meaning that it can be missed at the typical 3-month check-up [93].

### 6.11. Myocarditis

Myocarditis occurs in 0.1–1% of patients on ICIs, typically shortly after initiation, with a high mortality rate. Diagnosis with FDG PET might be difficult due to limited sensitivity (~30%) because of variable myocardial FDG uptake in healthy individuals [7]. There has been a case reported by Arponen et al. describing ICI-myocarditis, not seen in cardiac magnetic resonance imaging (MRI), with FDG PET that revealed diffuse activity in the left ventricle and a focal area with an SUVmax of 5.4 at the base of the septum [97].

Physiological FDG uptake is differentiated from pathological with the help of protocols that include diet and heparin to reduce background uptake [79,98]. A clinical trial conducted by Ederhy et al. [99] showed that FDG-PET did not have a significant value to detect ICI-myocarditis. This clinical trial used diet to suppress the physiological FDG uptake. In contrast to the above-mentioned trial, a study by Nensa et al. [100] showed that the use of a low carbohydrate diet in combination with unfractionated heparin significantly suppressed the physiological FDG uptake, thereby increasing the sensitivity and specificity of PET to detect ICI-induced myocarditis to 74% and 97%, respectively, in comparison to CMR. ^18^F-FDG PET/MRI (a hybrid imaging modality combining FDG PET with MRI) offers comprehensive myocardial assessment, valuable when biopsies are risky or other tests are inconclusive. Alternative PET tracers such as ^68^Ga-DOTATOC show promise for early detection of ICI-myocarditis, correlating with serum cardiac troponin I and immune markers, such as cytokines and chemokines [7].

ICI-pericarditis is less frequent but might cause cardiac tamponade leading to a cardiogenic state, with a mortality rate of 21%; it might co-occur with myocarditis [7].

### 6.12. Skin Reactions

Cutaneous irAEs are one of the most common types of irAEs, occurring in as many as 34% of patients receiving anti-PD-1/PD-L1 therapy and up to 45% of patients on CTLA-4 inhibitors [101]. FDG PET can identify conditions such as maculopapular rash, pruritus, or erythema nodosum, showing subcutaneous areas with enhanced tracer uptake or bilateral uptake under the orbits resembling black-eye stripes [102,103]. Still, in some types of cancer, e.g., melanoma, the reading physician should be alert to the FDG-avid skin nodules since they can mimic metastatic lesions.

### 6.13. Musculo-Skeletal

Arthritis has typically been controlled by ultrasound, MRI, or CT; however, FDG PET use has been growing in the field of arthritis, and it is even more sensitive in cases of soft tissue irAEs, such as synovitis, myositis, and fasciitis, where it shows diffuse periarticular uptake [104]. FDG PET patterns mimic rheumatoid arthritis (RA), polymyalgia rheumatica (PMR), psoriatic arthritis (PsA), and oligo-monoarthritis [105]. Ponce et al. (2022) categorized ICI-arthritis into self-limiting (>50%), intermittent (25%), or persistent chronic types (20%) [74,93,104,106]. A review by Cappelli et al. (2017) indicated that the incidence of the two most common irAEs, arthralgia and myalgia, was found in 1–43% and 1–21% of patients, respectively [54,107]. However, while FDG PET may be a sensitive method for diagnosing ICI-related musculo-skeletal disorders, other more available modalities, e.g., ultrasound, may be more cost-effective.

### 6.14. Soft Tissue irAEs

In rare cases, ICI therapy may cause panniculitis, involving retroperitoneal and subcutaneous fat, or fasciitis in the form of scleroderma-like eosinophilic changes (an uncommon irAE), manifesting on PET as fascial/fat hypermetabolism, with enhanced FDG uptake [108,109].

### 6.15. Encephalitis

FDG PET’s brain assessment is limited by natural gray matter uptake, but it can highlight irregularities in encephalitis cases. MRI is a preferable method for detecting autoimmune encephalitis due to its high sensitivity [109].

**Table 2:** Metabolic patterns of immune-related adverse events of immune checkpoint inhibitors in ^18^F-FDG PET/CT seen in literature.

irAE Type	Metabolic Activity and Patterns	Quantitative Data of Metabolic Patterns
Pneumonitis [7,11,29,54,55,79,81,82,83]	- Increased bilateral FDG uptake in the lungs, showing interstitial or peribronchovascular consolidations - Organizing pneumonia as focal or diffuse ground glass opacities - Bronchial wall thickening and micronodules	Mean SUV_max_: 3.36 (±1.7); Predominantly bilateral presentation.
Sarcoid-like reaction [6,10,11,83,90,91]	- Symmetrical enlargement of mediastinal and hilar lymph nodes - Bilateral perilymphatic lateral micronodules with consolidations	
Nephritis [7,11,54,79,93,96]	- >30% enlargement - Increased diffuse and bilateral uptake in the renal cortex,	- Increased parenchymal SUV_max_-to-blood pool ratio - Decreased renal pelvis SUV_max_-to-blood pool ratio
Colitis [11,29]	- Diffuse thickening of the colonic wall and pericolonic fat stranding - Segmental or diffuse increase in FDG uptake in the colon	
Arthritis [54,74,93,104,105,106]	- Heightened periarticular FDG uptake - RA-like, PMR-like, PsA-like and oligo-monoarthritis	
Hypophysitis [55,56]	- Increased pituitary to frontal SUV_max_ ratio, exceeding 0.3	Incidence: 3.25–14.0%
Pancreatitis [7,11,76,77,110]	- Diffusely increased FDG uptake in the pancreas resembling acute pancreatitis - Occasionally presents in a focal manner, mimicking metastases	
Thyroiditis [6,56,58,59,60,65,66]	- Diffuse thyroidal uptake of FDG	Peak thyroid ^18^F-FDG SUV_max_ uptake around day 142
Myocarditis [7,79,97,98]	- Diffuse increased activity in myocardium - Preparation protocols necessary before a dedicated cardiac PET	
Reactive splenomegaly [6,7,10,53,70,71,72]	- Increased spleen uptake and an inversion of the typical liver-to-spleen FDG uptake ratio	
Hepatitis [10]	- Increased liver-to-blood-pool SUV_mean_ ratio	healthy range: 1.3−1.4
Skin [7,103]	- Metabolically active subcutaneous nodules	
Gastritis [74]	- Increased localized or diffuse FDG uptake	
Esophagitis [54]	- Diffuse uptake along esophagus	
Encephalitis [109]	- Irregularities in the normal high physiological uptake in grey matter	

## 7. Pseudoprogression. Hyperprogression

**Figure 9:**
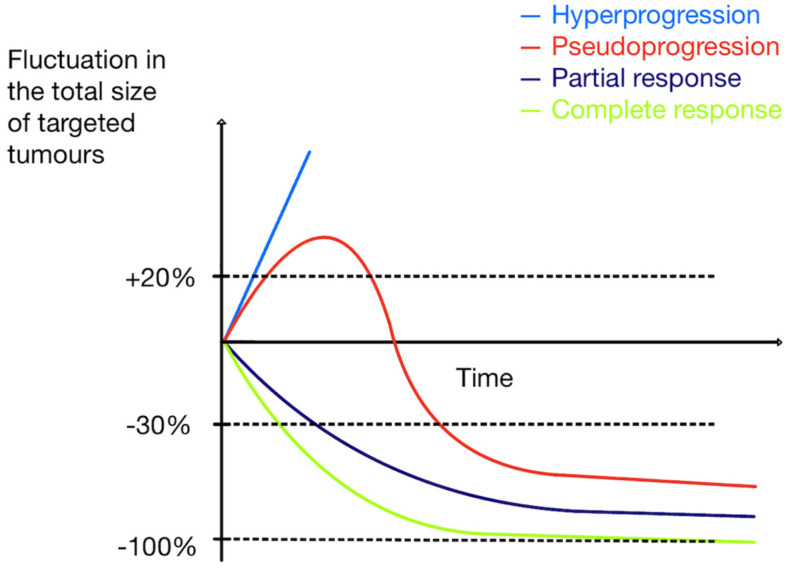
Patterns of tumor response during treatment. Adapted from Frelaut et al. [115].

*Pseudoprogression* is a reaction after initiating ICI therapy, with sudden tumor enlargement or new lesions seen on PET scans, followed by a decrease in tumor size/number on follow-up scans (an example in Figure 8). It is believed to be caused by immune cell infiltration and cytokine release. TILs consume glucose; therefore, tumor growth and further metabolic activation occur. Pseudoprogression is seen in up to 10% of metastatic melanoma patients treated with ICIs (15% with ipilimumab) and less frequently in other tumors [17,29,111,112,113,114]. It presents as an apparent progression on PET imaging (increase in size and/or FDG-avidity of existing lesions or increase in number of FDG-avid lesions) without clinical deterioration and followed by a response. Pseudoprogression usually takes place early during treatment. It is important to recognize it and not discontinue treatment prematurely before achieving clinical benefit. Figure 9 shows various patterns of tumor response during treatment. Adapted from Frelaut et al. [115].

*Hyperprogressive disease* (HPD) is a reaction occurring shortly aft er initiation of ICI therapy, mainly with anti-PD-1/anti-PD-L1, and appears as sudden rapid tumor growth, typically two-fold or higher, and relates to a worse clinical outcome, making it necessary to diagnose it early [29,103,107]. The incidence of HPD varies across different cancer types, reaching about 8–30% [108,109]. There is ongoing debate about HPD’s validity; some suggest it could occur independently of therapy [29]. The biological background of HPD is still not well understood. Risk factors are related to tumor biology (e.g., MDM2/MDM4 amplification or cancer type) and advanced age [116,117,118,119]. Probably, HPD is not dependent on ICI type. Some radiological models predicting HPD risk, incorporating FDG PET scans, could potentially enable the prediction of HPD, decreasing the risk of delayed treatment adaptation. Notable is that HPD is associated with a higher metabolic tumor burden at baseline PET/CT [114,116,120].

## 8. Summary. Future Perspectives

In oncology, FDG PET is used to monitor therapy effectiveness, adverse events, and detect non-responders, with high FDG uptake indicating more malignant tumors and being a negative prognostic factor [121]. During immunotherapy with ICIs, PET can detect irAEs with 83% sensitivity, as found by Girard et al. (2020) [122]. This review aims to elucidate various metabolic patterns of irAEs commonly detected in FDG PET during the course of ICI treatment. It should be kept in mind that FDG PET is not treated as a dedicated method for detecting and monitoring irAEs, but rather irAEs are revealed incidentally during routine control studies. Therefore, nuclear medicine physicians should be particularly vigilant to these signs to help clinicians effectively manage their patients.

In asymptomatic patients, the detection of abnormal FDG uptake via PET/CT scans suggesting irAEs may lead clinicians to consider preemptive actions to mitigate the progression to symptomatic irAEs. However, this must be approached with caution. While FDG PET/CT can provide crucial early warnings of irAEs, the method’s sensitivity might also lead to false positives—abnormal results that do not reflect clinically significant problems.

Premature discontinuation of ICI based on FDG PET/CT findings alone can deprive patients of an effective therapeutic regimen, potentially impacting their cancer prognosis adversely. The decision to halt immunotherapy should not rely solely on imaging results but should be confirmed by clinical assessment, laboratory markers, and a multidisciplinary team’s judgement. Such an integrated approach would allow us to balance the benefits of continued immunotherapy against the risks of serious irAEs, avoiding unnecessary cessation of treatment and suboptimal cancer control. Some studies even indicated that certain irAEs may be a good prognostic sign for ICI efficacy as they have been connected with increased progression-free survival and overall survival in certain situations [123]. To the authors’ best knowledge, there are currently no protocols that can be used for diagnosing irAE with PET; therefore, further research would be beneficial to create guidelines on interpreting FDG PET/CT results in the context of ICIs.

The joint practice guidelines of three nuclear medicine societies in Europe, the US, and Australia (i.e., EANM, SNMMI, and ANZSNM) recommend the use of FDG PET at three time points: Before initiation of ICI therapy serving as a baseline for tumor monitoring, 8–12 weeks after treatment start, complementary to the information obtained from anatomical imaging, and before immunotherapy discontinuation in patients receiving long-term treatment, especially in cases of partial response or stable disease on CT [124]. A limitation of FDG PET to serve as an optimal imaging modality for detecting irAEs is its unsatisfactory specificity and challenging differentiation between tumor progression. To a certain extent, this can be overcome by assessing the nature of the uptake (diffuse vs. focal) and the experience of the reading physician; however, occasionally invasive procedures may be necessary to establish a clear diagnosis. Also, wider clinical indications for PET would be an additional financial burden, with reimbursement issues already being a significant limiting factor across the countries. A summary of pros and cons of FDG PET in detecting irAEs is presented in Table 3.

In the era of rapidly developing molecular imaging, one can assume that new tracers may appear and will help understand the underlying pathophysiological mechanisms of tumor microenvironment and immune-mediated reactions. Recently, immuno-PET-enabling in vivo visualization of immune checkpoint inhibitors was presented [125]. With the advent of zirconium-89 (89Zr)-labeled nivolumab, pembrolizumab, atezolizumab, or durvalumab, it is now possible to determine the PD-1/PD-L1 expression in all tumor lesions throughout the body, without being limited to histopathological analysis of a single tissue sample. This opens enormous opportunities for personalized and precision medicine and, hopefully, the prediction of response to ICIs.

A limitation of this review is the fact that it is mostly based on studies including small numbers of patients, as well as case series and case reports. Since FDG PET is more and more commonly used in oncological follow-up, one can anticipate that the body of evidence, including randomized controlled trials, concerning the role of FDG PET in detecting irAEs will grow.

## 9. Conclusions

This review emphasizes the critical role of integrating ^18^F-FDG PET/CT as a sensitive method for detecting irAEs into the standard monitoring protocols for patients undergoing ICI therapy. This approach not only facilitates timely intervention for irAEs but also aids in optimizing treatment strategies, ultimately leading to improved patient outcomes. Future research should prioritize standardizing PET/CT protocols and defining clear criteria for both response assessment and irAEs detection. However, it is essential to acknowledge potential limitations in integrating FDG PET/CT into the routine clinical management of patients undergoing immunotherapy. These limitations may include challenges in distinguishing between irAEs and tumor progression, as well as reimbursement issues, which may also hinder more widespread use of the method. Addressing these issues will be crucial to realizing the full potential of FDG PET/CT in enhancing the care of patients treated with immunotherapy.

## Figures and Tables

**Table 1:** An overview of several malignancies commonly treated with ICIs (alone or in combination therapy) [15,17,19,22,23,24,25,26,27,28,29,30,31,32,33,34,35,36,37,38,39]. NSCLC—non-small cell lung cancer, SCLC—small cell lung cancer, MSI-H or dMMR CRC—Microsatellite-high or deficient mismatch repair colorectal carcinoma, BCC—Basal cell carcinoma, CSCC—cutaneous squamous cell carcinoma, ESCC—esophageal squamous cell carcinoma.

	Ipilimumab	Nivolumab	Pembrolizumab	Atezolizumab	Durvalumab	Avelumab	Cemiplimab	Dostarlimab	Retifanlimab	Toripalimab	Tremelimumab	Relatlimab	Fianlimab
Melanoma	X	X	X	X						X		X	X
Merkel cell carcinoma			X			X			X				
NSCLC	X	X	X	X	X		X				X		
SCLC		X	X	X	X								
Renal carcinoma		X		X		X							
Squamous cell carcinoma of head and neck		X	X	X	X								
Urothelial carcinoma		X	X	X	X	X							
Hepatocellular carcinoma		X		X							X		
Triple negative breast cancer				X									
MSI-H or dMMR CRC	X	X	X	X									
Hodgkin lymphoma		X	X										
BCC							X						
CSCC							X						
Cervical cancer			X				X						
Endometrial cancer								X					
Nasopharyngeal carcinoma										X			
ESCC		X	X										
Pleural mesothelioma	X	X											

**Table 3:** Pros and cons of ^18^F-FDG-PET/CT as an imaging modality for detecting irAEs related to ICI treatment.

Pros	Cons
**Early Detection of irAEs**—^18^F-FDG PET/CT can identify early signs of inflammation and metabolic changes sometimes indicating irAEs [13].	**High Costs and Accessibility**—The procedure is expensive and may not be accessible in all healthcare settings, which could limit its regular use [17].
**Comprehensive Coverage**—This imaging modality can monitor multiple organs simultaneously, crucial as irAEs can affect various systems, including the endocrine glands, liver, lungs, and more [122].	**Challenges in Differentiation**—It can be difficult to distinguish between signs of irAEs and tumor progression or other non-cancerous conditions, potentially leading to diagnostic ambiguity [7].
**Sensitive in Monitoring Progression and Response**—Besides detecting irAEs, ^18^F-FDG PET/CT is also highly effective in monitoring disease progression and response to therapy, which is essential for adjusting treatment plans [11].	**Radiation Exposure**—As with any imaging technique that involves radiation, there is an inherent risk of exposure, which must be managed, especially in scenarios requiring multiple follow-ups [18].
**Predictive Insights**—Changes in FDG uptake patterns may provide predictive insights into treatment efficacy and patient outcomes, contributing to personalized treatment strategies [10].	**Premature discontinuation of treatment**—False-positive results with PET could potentially lead to an unnecessary cessation of ICI [13].

## Data Availability

Data sharing is not applicable to this article.
